# High Dose Ifosfamide in Relapsed and Unresectable High-Grade Osteosarcoma Patients: A Retrospective Series

**DOI:** 10.3390/cells9112389

**Published:** 2020-10-31

**Authors:** Emanuela Palmerini, Elisabetta Setola, Giovanni Grignani, Lorenzo D’Ambrosio, Alessandro Comandone, Alberto Righi, Alessandra Longhi, Marilena Cesari, Anna Paioli, Rossella Hakim, Michela Pierini, Emanuela Marchesi, Daniel Vanel, Ymera Pignochino, Davide Maria Donati, Piero Picci, Stefano Ferrari

**Affiliations:** 1 Chemotherapy Unit, IRCCS Istituto Ortopedico Rizzoli, 40136 Bologna, Italy; emanuela.palmerini3@unibo.it (E.P.); elisabetta.setola@ior.it (E.S.); alessandra.longhi@ior.it (A.L.); marilena.cesari@ior.it (M.C.); anna.paioli@ior.it (A.P.); rossella.hakim@ior.it (R.H.); michela.pierini@ior.it (M.P.); stefanoferrari.19855@gmail.com (S.F.); 2 Division of Medical Oncology, Candiolo Cancer Institute, FPO-IRCCS. St. Provinciale 142, Km 3.95, 10060 Candiolo, Torino, Italy; giovanni.grignani@ircc.it (G.G.); ymera.pignochino@ircc.it (Y.P.); 3 Ospedale San Giovanni Bosco, 10154 Torino, Italy; alessandro.comandone@aslcittaditorino.it; 4 Pathology Unit, IRCCS Istituto Ortopedico Rizzoli, 40136 Bologna, Italy; alberto.righi@ior.it (A.R.); vanel.daniel@yahoo.fr (D.V.); 5 Italian Sarcoma Group, IRCCS Istituto Ortopedico Rizzoli, 40136 Bologna, Italy; emanuela.marchesi@italiansarcomagroup.org (E.M.); piero.picci@italiansarcomagroup.org (P.P.); 6 Orthopedic Oncology, IRCCS Istituto Ortopedico Rizzoli, 40136 Bologna, Italy; davide.donati@ior.it

**Keywords:** osteosarcoma, high-grade bone sarcoma, pediatric bone tumors, ifosfamide, chemotherapy, PARP1, sequencing

## Abstract

*Background*: The evidence on high-dose ifosfamide (HD-IFO) use in patients with relapsed osteosarcoma is limited. We performed a retrospective study to analyze HD-IFO activity. *Methods*: Patients with osteosarcoma relapsed after standard treatment [methotrexate, doxorubicin, cisplatin +/− ifosfamide (MAP+/−I)] with measurable disease according to RECIST1.1 were eligible to ifosfamide (3 g/m^2^/day) continuous infusion (c.i.) days 1–5 q21d. RECIST1.1 overall response rate (ORR) (complete response (CR) + partial response (PR)), progression-free survival at 6-month (6m-PFS), duration of response (DOR), and 2-year overall survival (2y-OS) were assessed. PARP1 expression and gene mutations were tested by immunohistochemistry and next-generation sequencing. *Results*: 51 patients were included. ORR was 20% (1 CR + 9 PR). Median DOR was 5 months (95%CI 2–7). Median PFS, 6m-PFS, OS, and 2y-OS were 6 months (95%CI 4–9), 51%, 15 months (10–19), and 30%, respectively. A second surgical complete remission (CR2) was achieved in 26 (51%) patients. After multivariate analysis, previous use of ifosfamide (HR 2.007, *p* = 0.034) and CR2 (HR 0.126, *p* < 0.001) showed a significant correlation with PFS and OS, respectively. No significant correlation was found between outcomes and PARP1 or gene mutations. *Conclusions*: HD-IFO should be considered as the standard first-line treatment option in relapsed osteosarcoma and control arm of future trial in this setting.

## 1. Introduction

High-grade osteosarcoma is the most frequent primary bone tumor that usually occurs in children and young adults [1,2]. Since the introduction of multi-agent chemotherapy (cisplatin, adriamycin, methotrexate, +/− ifosfamide) significant improvement in prognosis has been registered with a 5-year survival rate increase from 10% (surgery alone) to nearly 70% (surgery and chemotherapy) in patients with localized disease [3,4,5]. However, when recurrence or progression occur, systemic treatments fail to give substantial benefit and post-relapse survival remains poor: less than 30% at 5-year [5,6,7,8] with a clear benefit from the achievement of a surgical, second complete remission (CR2), whereas the benefit from systemic treatment is still on debate [3]. 

Ifosfamide (or cyclophosphamide) at standard or high dose alone or in combination with etoposide, and gemcitabine/docetaxel, have all been employed for patients with osteosarcoma at the time of recurrence [9,10,11,12,13,14,15,16,17,18] (Table 1). 

At present, high dose ifosfamide (HD-IFO) is largely used at the time of recurrence in osteosarcoma. Several studies are available on this regimen with doses up to 12–15 g/m^2^ in patients with relapsed osteosarcoma. Interestingly, they mainly focused on response rate whereas clinically relevant information such as PFS rate and variables influencing response and survival are lacking [18,19,20,21] (Table 1). We performed a retrospective study to assess the clinical activity of HD-IFO in patients with relapsed and unresectable osteosarcoma after standard treatment of their localized disease and explored its correlation with key molecular features.

## 2. Patients and Methods

Patients with high-grade osteosarcoma who received chemotherapy with HD-IFO when relapsing after standard treatment were selected from the database of the Rizzoli Institute. Only patients with measurable disease according to RECIST 1.1, deemed not suitable for upfront surgery after multidisciplinary team (MDT) evaluation (including thoracic surgeons, orthopedics, radiologists, radiation therapists and medical oncologists), who received at least two courses of HD-IFO, and with available demographic, clinical, imaging and follow-up data were included in the present analysis. This study was approved by the local ethical committee. Demographic and clinical data (age, sex, ECOG scale of performance status, pattern of relapse, type, number and prior treatments (metastasectomy before HD-IFO and histologic response on primary tumor after induction chemotherapy), number of HD-IFO courses, radiologic response according to RECIST 1.1, toxicity, date of progression, type of treatment after HD-IFO, date of last follow-up or death) were collected from patient chart. Radiological images were reviewed (D.V., P.P. and E.P.) for the purpose of this study. 

Treatment consisted of ifosfamide 3 gr/m^2^/day combined with MESNA at same dose, given as continuous infusion from day 1 to 5, every 21 days [22], until progression or unacceptable toxicity. Prophylactic use of G-CSF was recommended from day 7 of each cycle.

According to institutional guidelines, response to HD-IFO was assessed by means of CT scan every two cycles according to RECIST criteria version 1.1. The achievement of a complete surgical excision of all metastases (second complete surgical remission = CR-2) was the mainstay of the treatment and surgery was offered to selected patients in case of partial response or stable disease, after multidisciplinary team discussion. Archival formalin-fixed paraffin-embedded (FFPE) tumor tissue were analyzed by standard immunohistochemistry procedure using DAKO Autostainer (Agilent, Santa Clara, CA, United States) and recombinant Anti-PARP1 antibody (E102, ab32138, Abcam, Cambridge, MA, United States). An expert pathologist evaluated PARP1 expression in tumor nuclei and scored as positive (+) tumor samples with more than 50% expressing tumor nuclei in three different optical fields. Visible images were acquired with a DM1000 microscope (Leica, Wetzlar, Germany) equipped with a color 3.1 M PixelCMOS digital camera. Tumor areas were microdissected from 10 µM slices and nucleic acids were extracted by Maxwell RSC DNA FFPE Kit and Maxwell RSC RNA FFPE Kit using a Maxwell RSC Instrument (Promega, Madison, WI, United States), according to manufacturer’s instructions. DNA and RNA were quantified using a Qubit^®^ 3.0 Fluorometer (Thermo Fisher Scientific, Life Technology Italia, Monza, Italy) and Qubit dsDNA HS (high sensitivity) assay kit (Thermo Fisher Scientific, Life Technology Italia). DNA and RNA quality were assessed by gel electrophoresis and 2100 Bioanalyzer High Sensitivity DNA and RNA assay Kits (Agilent Technologies, Agilent Technologies, Inc., Santa Clara, CA, USA). Nucleic acids purity was evaluate using NanoDropTM (Thermo Fisher Scientific). Ten ng of QC-passing nucleic acid samples were loaded on IonChefTM System for library preparation according to Oncomine Comprehensive Cancer panel assay v3 TM (Thermo Fisher Scientific). In the presence of IonCodeTM barcodes, eight 100 pM prepared DNA and RNA libraries were diluted to 50 pM, pooled and the single stranded template libraries and loaded on the Ion 540™ chip by IonChefTM System. Sequencing was achieved using the Ion GeneStudioTM S5 Plus System. The panel results were optimized for a total of 8 to 10 million reads with a 500× median coverage. The reads were aligned to assembly hg19 of the human reference genome by the Torrent SuiteTM (v 5.8). To standardize the analyses and to reduce the impact of sequencing artifacts derived from the formalin fixation, we set the allele frequency limit of detection at 10% for all the samples.

Toxicity data were collected both from clinical chart and from a “toxicity data form” filled by patients or their guardians. Toxicity data were analyzed and graded according to the Common Toxicity Criteria for Adverse Events (CTCAE) version 4.03. 

Primary endpoint was overall response rate (ORR) [defined as complete response (CR) + partial response (PR)], according to RECIST 1.1. Progression-Free survival (PFS), PFS at 6-month (6m-PFS) defined as the ratio between patients in CR, PR or stable disease (SD) and those ones progressing (PD) after six months from study entry. Overall survival (OS), OS at 2-year and duration of response and toxicity were secondary end-points of the study. Patients who underwent radical surgery after HDIFO were defined as CR2.

PFS was calculated from the date of the first cycle of HD-IFO to the date of tumor progression or last follow-up. Duration of response was calculated from first day of chemotherapy (HD-IFO) to the date of tumor progression. Overall survival (OS) was calculated from the date of the first day of HDIFO chemotherapy to the date of death or last follow-up (post-HDIFO survival). Patients were censored at the date of last follow up in the absence of death or progression. 

Statistical analyses were performed using SPSS v26.0 (IBM, Armonk, NY, United States). We used descriptive statistics for baseline patients’ characteristics. Qualitative variables were compared using the χ^2^ and Fisher’s exact tests and/or the Mantel-Haenszel odds ratio (OR) estimates. Survival endpoints were computed by Kaplan-Meier method. Log-rank test and hazard ratio (HR) estimates calculated by Cox regression were used for comparisons. Multivariate analysis was performed using the Cox proportional hazards model including covariates with *p*-value ≤ 0.05 in the univariate analysis. Whenever indicated, tests were performed two-sided and results were reported with 95% confidence intervals (95%CI). We considered a *p*-value ≤ 0.05 as statistically significant.

## 3. Results

Fifty-one patients treated with HD-IFO from 1992 to 2014 were identified. Clinical characteristics are reported in Table 2. Median age was 19 (range 8–68). Twenty-one (41%) patients were pediatric (<18 years-old). All patients were pretreated with doxorubicin (cumulative dose ranging from 360 to 420 mg/m^2^) and cisplatin (cumulative dose of 600 mg/m^2^), while methotrexate (cumulative dose 36–120 g/m^2^) was administered to all patients younger than 40 years. Thirty-two (63%) patients had already been treated with standard dose of ifosfamide for the primary tumor, according to the following schedules: ifosfamide 2 g/m^2^/day, day 1–5, 5 day-continuous infusion, Ifosfamide 3 g/m^2^/day, day 1–3, or Ifosfamide 2.5 g/m^2^/day, day 1–3 (cumulative dose 30–50 gr/m^2^). Twenty-six (51%) patients had undergone previous lung metastasectomy at the time of their first recurrence.

In 46 (90%) patients, HD-IFO was administered as first-line treatment, in 4 (8%) patients as second-line and in 1 (2%) patient in third-line (after failure of two previous lines of chemotherapy for metastatic disease). Agents administered after failure of HDIFO were: gemcitabine/docetaxel in seven cases (14%), sorafenib/everolimus in seven (14%), cyclophosphamide/etoposide in six (12%), methotrexate in three cases (6%), high-dose chemotherapy with peripheral blood stem cell support (+ samarium 153 in two) in three (6%) cases, oral etoposide (+ oral metothrexate in one) in three (6%) patients, pemetrexed in two (4%), and cisplatin, carboplatin, interferon-α, oral methotrexate/ cyclophosphamide, trabectedin, gemcitabine monotherapy in one (2%) case each. 

### 3.1. Response

All 51 patients received at least two courses of chemotherapy and were evaluable for response. One (2%) patient achieved a CR, 9 (18%) patients a PR for an ORR of 20%, with a median of 2 cycles to best response (range 2–4) (Table 3). The median duration of response was 7 months (range 2–192). The ORR was 29% (1 CR + 5 PR) and 13% (4 PR) (OR 2.60 95%CI 0.631–10.711, *p* = 0.186) in the pediatric and adult population, respectively (Table 3). 

### 3.2. Progression Free Survival (PFS)

**Figure 1:**
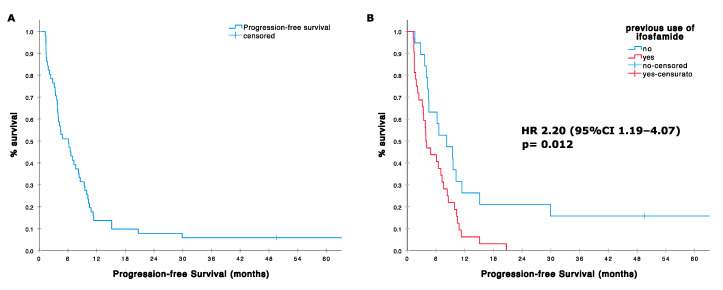
(**A**) Progression-free survival (PFS) in 51 patients treated with high dose ifosfamide; (**B**) PFS according with prior use of ifosfamide standard dose in the adjuvant setting.

The median PFS was 6.1 months (95%CI 3.7–8.5; range 1–195 months). The 4- and 6-month PFS were 61% (95%CI; 47–75) and 51% (95% CI, 37–65), respectively. PFS was significantly better in the group of patients treated in first line compared with patients treated in second or further line (median 6.2 months, 95%CI 2.8–9.7 vs. 3.1, 95%CI 0–6.8; HR 0.369, 95%CI 0.141–0.966, *p* = 0.042, Figure 1, Table 4). Interestingly, poor histological response to former induction chemotherapy was not associated with significant inferior PFS (HR 0.939, *p* = 0.833). On the contrary, prior standard-dose ifosfamide treatment negatively affected PFS (median 3.9, 95%CI 2.3–5.4 vs. 8.2, 95%CI 3.7–12.7; HR 2.20, 95%CI 1.19–4.07, *p* = 0.012). No significant differences were observed according to age, sex, ECOG, and pattern of metastases.

### 3.3. Overall Survival (OS) Post HDIFO

**Figure 2:**
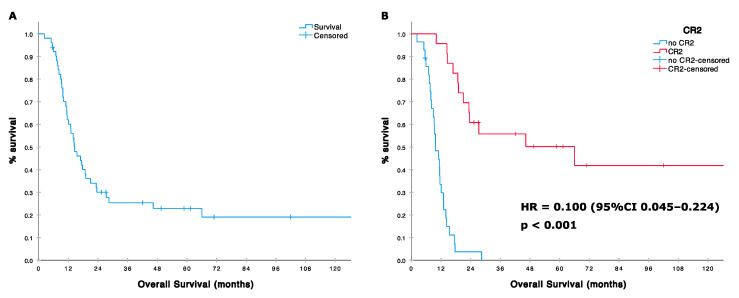
(**A**) Overall survival (OS) in 51 patients treated with high dose ifosfamide; (**B**) OS according with surgical, second complete remission (CR2) status.

The median post HD-IFO survival was 14.5 months (95%CI 10.1–18.9; range 2–260 months). 1-year and 2-year OS were 62% (95%CI 49–75) and 30% (95%CI 19–45), respectively (Figure 2). Patients reaching CR/PR and SD had a 2-year OS of 44% and 38%, respectively, whereas none was alive at 2 years in case of PD (HR for OS in patients achieving at least a disease stabilization vs. progression patients was 0.223, 95%CI 0.106–0.470 *p* < 0.001; Table 5). Twenty-three responding patients (including patients with tumor shrinkage not reaching the RECIST 1.1 threshold for defining a partial response) underwent metastasectomy, achieving a CR2 status. Twenty-two patients had lung metastases (with concomitant local recurrence in three patients). One had a synchronous bone lesion. Patients achieving a CR2 had a significantly longer survival compared to others with a median OS of 66 (95%CI 2–130) vs. 10 months (95%CI 7–13) and an HR for death of 0.100 (95%CI 0.045–0.224, *p* < 0.001). Two-year OS in the group of patients achieving a CR2 was 61% vs. 4% in patients who did not (Figure 2). Prior treatment with standard-dose ifosfamide was associated with a poorer prognosis (median OS 12, 95%CI 6–18 vs. 23 95%CI 0–62; HR 2.339, 95%CI 1.159–4.719, *p* = 0.018), while former good histologic response to primary chemotherapy (necrosis ≥ 90%) showed a nearly significant correlation with improved survival [median OS 19 (95%CI 15–22) vs. 13 months (95%CI 9–17); HR 0.527 (95%CI 0.271–1.025), *p* = 0.059]. Table 5.

### 3.4. PFS and OS Multivariate Analysis

After multivariate analysis, patients with no prior use of ifosfamide (HR 0.498, 95%CI 2.62–0.947, *p* = 0.034) and achieving a CR2 status (HR 0.126, 95%CI 0.053–0.299, *p* < 0.001) had a significant improvement in PFS and OS, respectively.

### 3.5. Toxicity

All the 51 patients were evaluable for safety analysis. The median number of cycles administered was four (range two to seven). Nine (18%) patients experienced a febrile neutropenia. Grade 3–4 anemia in two (4%) patients and thrombocytopenia in three (6%) cases was reported, with one patient interrupting treatment due to thrombocytopenia. Grade 3–4 neurological toxicity was described in two (4%) of the patients. In both cases neuropathy resolved after chemotherapy interruption, hydration, diuretics and methylene blue. Two patients (4%) experienced a grade 1 persistent kidney injury. Subsequent dose reduction was required in all patients with non-hematological toxicity. 

### 3.6. PARP1 Expression and Mutational Analysis

Twenty of 24 available FFPE tumor samples were immunohistochemically evaluable for PARP1 expression. This was mostly due to deterioration of tumor samples related to decalcification and we were able to perform adequate analyses mainly in patients for whom tumor samples from metastases were available. Thirteen of 20 (65%) displayed high PARP1 expression (Supplementary Table S1). High-PARP1 expression showed a trend toward a worse outcome without reaching statistical significance. Indeed, median PFS was 2.4 (95%CI 1.0–3.9) vs. 9.4 months (95%CI 1.8–17.0) (HR 1.79, 95%CI 0.68–4.72 *p* = 0.240); 6m-PFS was 38.5% vs. 71.4% (OR 0.25, 95%CI 0.03–1.82, *p* = 0.171), median OS was 13.0 (95%CI 1.6–24.5) vs. 14.5 months (95%CI 6.0–23.0) (HR 1.09, 95%CI 0.40–2.98 *p* = 0.859); and ORR was 14.3% vs. 15.4% (OR 1.091, 95%CI 0.08–14.7; *p* = 0.948) in patients with high- vs. low-PARP1 expression, respectively. Twelve of 24 patients’ samples had adequate nuclei acid extraction and were analyzed by targeted NGS. Two out of 12 (16.6%) evaluable patients showed oncogenic hotspot single nucleotide variation (SNV) on TP53 gene and KRAS genes, while 2/12 (16.6%) showed oncogene (MYC and CCNE1) amplifications (Supplementary Table S1). We performed exploratory analyses looking for correlations between ORR, PFS and OS and identified gene mutations. Indeed, in patients with gene mutation detected vs. others, median PFS was 7.0 (95%CI 0–15.2) vs. 2.4 months (95%CI 0.1–4.7) (HR 0.30, 95%CI 0.06–1.47 *p* = 0.138); 6m-PFS was 75% vs. 37.5% (OR 5.00, 95%CI 0.34–72.77, *p* = 0.239); median OS was 9.2 (95%CI 0–28.9) vs. 14.5 months (95%CI 0.6–28.5) (HR 0.63, 95%CI 0.16–2.52 *p* = 0.516); and ORR was 12.5% vs. 25.0% (OR 2.33, 95%CI 0.11–50.98.7; *p* = 0.590), respectively. 

## 4. Discussion

In this series a treatment with HD-IFO (15 g/m^2^) in recurrent/progressive osteosarcoma after standard treatment showed an ORR of 20% and up to nearly 30% in pediatric patients. The 6m-PFS was 53% and was significantly higher for whom were ifosfamide-naïve and treated in 1st line. The 2-year OS was 52% and markedly influenced by the high proportion of patients who achieved a second complete surgical remission that also in our series was confirmed as the most relevant factor influencing survival. 

In this setting only few studies, with small number of patients [16,17,18,19,20,21,22,23,24,25,26,27,28,29,30], and with different chemotherapy regimens, reported an ORR ranging from 0 to 30% [10,11,12,13,14,15,16,17,18,19,20]. Despite the potential ORR overestimation related to the inclusion of patients who received at least two cycles of HD-IFO, the results observed in our series are consistent with the three studies that reported on high dose (12–14 g/m^2^) ifosfamide, with an ORR ranging from 10 to 62% [24,25,26]. These data further support the use of this strategy in advanced osteosarcoma. Indeed, in similar settings and with the obvious limitation of different populations, several studies on tyrosine kinase inhibitors in advanced osteosarcoma have been published, displaying only modest activity in terms of tumor shrinkage: 9% with sorafenib [23], 7.7% to 14% for regorafenib [24,25], 7.7% with lenvatinib [26] and 14% with cabozantinb [27]. 

Interestingly, in our study ORR in pediatric patients was 29% vs. 13% in the adult patients (*p* = 0.186), with no difference both in PFS and in OS figures in the two different populations, confirming that age *per-se* does not represent a prognostic factor after relapse in osteosarcoma [8].

Beyond the possibility of prolonging disease control, a major goal of chemotherapy in osteosarcoma metastatic setting is to increase the chance of obtaining a radical surgery and/or delay further progression. In this series, in 23/51 non-progressing patients (including patients with CR/PR and those ones experiencing a tumor shrinkage < 30%) a CR2 status was achieved. Consistently with large previous experiences [7,8], the CR2 status was confirmed also in our series as a key factor associated with an improved survival. Due to the retrospective nature of our study, it is not possible to definitely determine the role of HD-IFO in CR2 achievement. Nonetheless, all the 23 patients who achieved a CR2 were considered not eligible to surgical resection at time of HD-IFO start by an experienced MDT. 

A dose-response mechanism is well-known for ifosfamide when used in soft tissue sarcoma [28]. Importantly, HD-IFO activity was observed also in patients previously treated with standard dose ifosfamide (from 6 to 10 g/m^2^), suggesting that higher doses could overcome at least some of the resistance mechanisms. Similarly, we observed some degree of activity of HD-IFO also in patients who received previous ifosfamide, but the PFS of these patients was significantly worse compared with those ifosfamide-naïve. This result could at least in part be related to both selection bias of poor prognostic features (in most of the of the studies adjuvant ifosfamide was given in case of poor histologic response) and chemo-resistance itself. This is in contrast with lack of association with PFS and histologic response to former induction chemotherapy. Taken together these findings, in view of EURAMOS1 clinical trial results [6], discourage use of adjuvant ifosfamide, unless within a clinical trial.

Of course, HD-IFO regimen is more toxic compared to ifosfamide given at lower doses (9–10 g/m^2^) in terms of both kidney and central neurological adverse events. Nevertheless, in our experience it was manageable, especially in the pediatric population, and no toxic death was reported in our series. Grade 3 or 4 of hematological side effects were reported in 14% of the patients overall; this rate might reflect under-reporting bias due to the retrospective design of this study. Febrile neutropenia occurred in nine patients (18%) in our study. Consistently, Verspor et al., described febrile neutropenia in 7 (19%) cases in a similar series with ifosfamide given from 6 gr/m^2^ to 9 gr/m^2^ [18]. Our series confirms that grade 3–4 neurological toxicity should be expected in at least 4% of the patients. Full recovery was achieved in both patients with hydration, diuretics and methylene blue, as described [29].

Molecular markers enabling clinical practices with predictive or prognostic valuable tools are warranted to better define patients who might really benefit from this treatment. PARP1 had shown predictive and prognostic values in bone and soft tissue sarcomas [30,31,32]. Moreover, peculiar gene defects showed implications in response to chemotherapy [33,34]. Unfortunately, in our retrospective case series paucity of available samples impinged on predictive and prognostic evaluation of PARP-1 and genetic abnormalities found on response to HD-IFO. Further prospective evaluation of this markers is needed to define their role, if any, in predicting prognosis and/or response to chemotherapy.

## 5. Conclusions

In conclusion, there are few new active regimens for patients with relapsed osteosarcoma following multimodality therapy. To our knowledge, this is the largest study on high-dose ifosfamide in pre-treated relapsed high-grade classic osteosarcoma, showing a relatively high ORR especially in pediatric patients. Our data support the use of this regimen as a first option for the treatment of metastatic disease, especially in oligometastatic cases. In this setting any tumor shrinkage making patients eligible to a second surgical complete remission raises chance of cure to about 25%. Finally, this study might be used as a benchmark for phase II studies in the setting of relapsed osteosarcoma.

## Figures and Tables

**Table 1:** Second-line option in patients with relapsed osteosarcoma.

Drugs	No	CR + PR	ORR	Authors
IFO 2 g/m^2^/day 1–3 ETO 100 mg/m^2^/day 1–3	32	2/3	16	Kung FH, Cancer 1992 [9]
IFO 2 g/m^2^/day 1–3 ETO 100 mg/m^2^/day 1–3	8	0/3	37	Miser JS, J Clin Onc 1997 [10]
CTX 500 mg/m^2^/day 1–5 ETO 100 mg/m^2^/day 1–5	14	1/3	28	Rodriguez-Galindo C, JPHO 2002 [11]
CTX 4000 mg/m^2^/day ETO 200 mg/m^2^/day 1–3	26	2/3	19	Berger M, Cancer, 2009 [12]
GEM 900 mg/m^2^/day, d1,8 TAXO 80–100 mg/m^2^, d1	14	0/1	7	Fox E. SARC 003, Oncologist 2012 [13]
GEM 675 mg/m^2^, d 1,8 TAXO 75–100 mg/m^2^, d1	10	0/3	30	Navid F, Cancer 2008 [14]
GEM 675–900 mg/m^2^, d 1,8 TAXO 100 mg/m^2^, d1	17	3/1	24	Song BS, Pediatr Blood Cancer 2014 [15]
GEM 675–900 mg/m^2^ d1,8 TAXO 75 mg/m^2^ d1	34	0/5	15	Palmerini E, BMC Cancer 2017 [16]
IFO 2.8 g/m^2^/day 1–5	23	nr	nr	Chou AJ, Cancer 2005 [17]
IFO 2.5 gr/m^2^ 1–2 or IFO 3 gr/m^2^ 1–3	26 36	6 13	23 36	Verschoor AJ, Oncologist, 2019 [18]
IFO 14 g/m^2^ CI d 1–14	19	2/6	42	Patel SR, JCO, 1997 [19]
IFO 14 g/m^2^ CI d 1–14	16	6/4	62	Berrak SG, Ped Blood Cancer 2005 [20]
IFO 12 g/m^2^ CI d 1–14	30	1/2	10	Harris MB, Med Ped Oncol 1995 [21]

%ORR: overall response rate; CR: complete response; PR: partial response; JPHO: Journal of Pediatric Hematology and Oncology; nr: not reported.

**Table 2:** Clinical characteristics, pattern of metastases and previous treatments in 51 patients treated with high-dose ifosfamide (HDIFO).

	*n*	%
All	51	100
Age median, range (years)	19 (7–68)	
≥18 years	30	59
<18 years	21	41
Sex		
male	33	65
female	18	53
ECOG		
0	47	92
1	4	8
Line at HDIFO		
1	46	90
≥2	5	10
Histology		
Osteoblatstic	38	74
Chondroblastic	6	12
Other	7	14
Metastases at diagnosis or relapse		
Diagnosis	5	90
Relapse	46	10
Median time from diagnosis to HD-IFO		
<24 months	29	57
≥24 months	22	43
Pattern of metastases		
Lung	35	68
Bone	3	6
Multiple sites	13	26
Ifosfamide in pretreatment		
yes	32	63
no	19	37
Previous metastasectomy		
yes	26	51
no	25	49
Primary tumor histologic response		
<90%	23	45
≥90%	24	47
Not available	4	8

pts = patients, HDIFO = high-dose Ifosfamide.

**Table 3:** Responses by RECIST 1.1 and age group in patients treated with high-dose ifosfamide.

	All (*n* = 51) *n* (%)	<18 yrs (*n* = 21) *n* (%)	≥18 yrs (*n* = 30) *n* (%)	*p*
CR PR	10 (20)	6 (28)	4 (13)	0.186
SD	29 (57)	10 (48)	19 (63)	
PD	12 (23)	5 (24)	7 (23)	

CR: complete response; PR: partial response; SD: stable disease; PD: progression of the disease.

**Table 4:** Univariate analysis for progression-free survival (PFS) from HDIFO start in patients affected by relapsed osteosarcoma.

	*n* (%)	Median Months (95%CI)	HR (95%CI)	*p* Value
All	51 (100)	6.1 (3.7–8.5)		
Age median, range (years)	19 (7–68)			0.540
≥18 years	30 (59)	6.1 (3.1–9.1)	0.835 (0.468–1488)	
<18 years	21 (41)	4.9 (0.47–9.3)		
Sex				0.789
male	33 (65)	4.9 (2–7.7)	1.086 (0.594–1.983)	
female	18 (53)	6.1 (1.5–10.7)		
ECOG				0.671
0	47 (92)	6.1 (3.4–8.8)	1.291 (0.397–4.196)	
1	4 (8)	7.3 (0.2–14.4)		
Line of CT				0.042
1° line	46 (90)	6.1 (3.0–9.2)	0.369 (0.141–0.966)	
≥2° line	5 (10)	3.1 (0–6.8)		
Pattern of metastases				0.890
Lung	35 (69)	6.5 (3.7–9.2)	0.957 (0.515–1.780) **	
Bone	3 (6)	1.3 (NE)		
Multiple sites	13 (25)	3.8 (3.2–4.4)		
Ifosfamide in pretreatment				0.012
yes	32 (63)	3.9 (2.3–5.4)	2.20 (1.19–4.07)	
no	19 (37)	8.2 (3.7–12.7)		
Histologic response for				0.833
primary *				
good	24 (51)	6.2 (2.9–9.5)	1.065 (0.592–1.918)	
poor	23 (49)	6.1 (2.8–9.4)		

PR: partial response; CR: complete response; SD: stable disease; PD: progression of the disease; NE not estimated. * not available in 4 cases, ** HR was computed comparing patients with lung metastases only vs. others.

**Table 5:** Univariate analysis for overall survival (OS) from HDIFO start in patients affected by relapsed osteosarcoma.

	*n* (%)	Median Months (95%CI)	HR	*p* Value
All	51 (100)	14.5 (10.1–18.9)		
Age median, range (years)	19 (7–68)			0.626
≥18 years	30 (59)	16.8 (11.8–21.9)	0.852	
<18 years	21 (41)	11.0 (5.4–16.6)	(0.448–1.620)	
Sex				0.721
male	33 (65)	15.4 (10.9–20.0)	0.885	
female	18 (53)	11.3 (10.4–12.2)	(0.454–1.728)	
ECOG				0.86
0	47 (92)	15.4 (10.6–20.3)	1.137	
1	4 (8)	13.0 (1.6–24.4)	(0.272–4.749)	
Line of CT				0.21
1° line	46 (90)	14.4 (10.2–18.6)	0.51	
≥2° line	5 (10)	9.3 (0–19.5)	(0.177–1.464)	
Response to CT				<0.001
CR/PR	10 (20)	14.4 2–26.8)		
SD	29 (57)	18.8 (8.6–29.0)	0.223	
PD	12 (23)	7.6 (3.4–11.9)	(0.106–0.470) **	
Pattern of metastases				0.484
Lung	35 (69)	16.8 (12.6–21.1)		
Bone	3 (6)	17.7 (NE)	0.788	
Multiple sites	13 (25)	9.8 (6.9–12.7)	(0.404–1.537) ***	
Prior ifosfamide				0.018
no	19 (37)	23.5 (0–62.4)	0.428	
yes	32 (63)	12.1 (.0–18.1)	(0.212–0.863)	
Histologic response for				0.059
Primary *				
good	24 (51)	18.8 (15.4–22.2)	0.527	
poor	23 (49)	13.0 (8.6–17.5)	(0.271–1.025)	
CR2				<0.001
yes	23(45)	66.0 (1.7–130.4)	0.100	
no	28 (55)	9.8 (7.0–12.6)	(0.045–0.224)	

PR: partial response; CR: complete response; SD: stable disease; PD: progression of the disease; CR2 surgical complete remission after HDIFO; NE not estimated. * not available in 4 cases, ** HR was computed comparing patients achieving at least a disease stabilization vs. progressing patients. *** HR was computed comparing patients with lung metastases only vs. others.

## Supplementary Materials

The following are available online at https://www.mdpi.com/2073-4409/9/11/2389/s1, Table S1: Genomic alterations and PARP1 expression in 24 tumor samples from 51 patients treated with high-dose ifosfamide (HDIFO).
