# Supplementary material for: High Dose Ifosfamide in Relapsed and Unresectable High-Grade Osteosarcoma Patients: A Retrospective Series
Source: Cells. 2020 Oct 31;9(11):2389. doi: 10.3390/cells9112389 (PMC7692098; doi:10.3390/cells9112389)
Supplement: Supplementary file 1 [file cells-09-02389-s001.pdf]

Supplementary Table S1

Genomic alterations and PARP1 expression in 24 tumor samples from 51 patients treated with high-dose ifosfamide (HDIFO)

	Gene alteration* (allele frequency, copy number)	COSMIC/ClinVar‡	PolyPhen-2φ	%PARP1 positive tumor cells§
met OS1	n.e.			65
met OS2	WT			60
met OS3	TP53 Leu194Phe (15%)	200/Likely Pathogenic	Probably damaging score 1.00	60
met OS4	n.e.			n.e.
met OS5	WT			60
met OS6	KRAS Gly12Val (31%)	10690/Pathogenic	Probably damaging score 1.00	35
met OS7	WT			80
met OS8	n.e.			40
met OS9	n.e.			n.e.
met OS10	n.e.			30
met OS11	n.e.			35
met OS12	WT			75
met OS13	n.e.			n.e.
met OS14	WT			30
met OS15	n.e.			60
met OS16	WT			60
met OS17	n.e.			55
met OS18	WT			65
met OS19	n.e.			n.e.
met OS20	MYC ampl (11 copies); CCNE1 ampl (18 copies)	856/Pathogenic 312/ Pathogenic		90
met OS21	n.e.			65
met OS22	n.e.			25
met OS23	WT			65
met OS24	MYC ampl (9 copies)	856/Pathogenic		70

* Oncomine Comprehensive Cancer panel v3 genes

‡ Number of observation of the variations as reported in COSMIC database /Significance of the genomic variation in relation to human health

φ Prediction of functional effects of human nsSNPs and probability score

§ % of PARP1 positive nuclei after conventional immunostaining and counting on 4 different optical microscopy field by an expert pathologist

WT: wild type; n.e.: not evaluable, the sample did not reach the quality score required for the analysis, insufficient starting material or purity of extracted DNA
